# Discrepancy between the Status Quo and Adjusted Risk of First-Onset Suicidal Ideation in Older Adults: A Longitudinal Study Based on the Korean Welfare Panel Study (2011–2021)

**DOI:** 10.3390/jcm12010224

**Published:** 2022-12-28

**Authors:** Youngdae Cho, Suk-Yong Jang, Eun-Cheol Park, Jean Kyung Bak

**Affiliations:** 1 Department of Public Health, Yonsei University Graduate School, Seoul 03722, Republic of Korea; 2 Division of Health Insurance Benefits, Ministry of Health and Welfare, Sejong 30113, Republic of Korea; 3 Department of Healthcare Management, Graduate School of Public Health, Yonsei University, Seoul 03722, Republic of Korea; 4 Department of Preventive Medicine, Yonsei University College of Medicine, Seoul 03722, Republic of Korea; 5 Institute of Health Services Research, Yonsei University, Seoul 03722, Republic of Korea; 6 Jeju Institute of Public Health & Health Policy, Jeju 63241, Republic of Korea

**Keywords:** older adults, middle aged, suicidal ideation, suicide, incidence

## Abstract

Whether older adults can more likely commit suicide than those in other age groups, after adjusting for other possible causes, remains unknown. We aimed to examine why elderly individuals are more likely to develop first-onset suicidal ideation than individuals in other age groups. We identified 2018 young, 3329 middle-aged, and 2714 elderly individuals without a history of suicidal ideation, from the Korean Welfare Panel Study 2011–2021. To determine key stressors that can induce suicidal ideation, selected groups of variables were adjusted progressively in a generalized estimating equation (GEE) model. Incidence rates of the elderly, middle-aged, and young individuals were 15.9, 22.0, and 11.3 per 1000 person-years, respectively. In GEE analysis, a positive association was not noted between age group and suicidal ideation after adjusting for stressor variables. Furthermore, the overly adjusted model (Full model) showed a strong negative association with aging; young [odds ratio (OR): 1.68, 95% confidence interval (CI): 1.35–2.11] and middle-aged (OR: 1.94, 95% CI: 1.38–2.73) individuals were more likely to develop first-onset suicidal ideation than the elderly. We found that full models, particularly assessing wealth rather than income, can explain why the elderly have higher suicide rates than those in other age groups.

## 1. Introduction

South Korea is ranked at a high level regarding the accessibility of healthcare services among the other Organisation for Economic Co-operation and Development (OECD) countries [1]. However, suicide rates in South Korea have been increasing since the mid-to-late 1990s, coinciding with the worldwide economic crisis during that time. In 2019, among the member states of the OECD, South Korea had the highest suicide rate at 24.6 per 100,000 individuals, which was more than twice the average of 12.0 for the other OECD countries [2]. In 2020, suicide was the first, second, fourth, and fifth leading cause of death in South Korea for those aged 0–39, 40–59, 60–69, and the entire population, respectively [3]. Although the overall suicide rate in South Korea declined slightly in 2020 to 25.6 per 100,000 persons from its highest point in 2011, the general trend is not as pronounced as the falling rates of suicide in other OECD countries [2,3]. In 2020, the suicide rates of those in the age groups of 60–69, 70–79, and over 80 were 30.1, 38.8, and 62.6 per 100,000 people, respectively; these rates were over 10% higher than the suicide rates of 21.7 and 27.1 per 100,000 people for those in the age groups of 20–29 and 30–39 years [3]. Poverty and the low socioeconomic status of the elderly may increase the risk of suicidal behaviors in South Korea [1].

However, since most prior studies have scrutinized factors associated with suicide in a specific age group (e.g., the older adults) or an entire nation [4,5,6,7,8], it is not known whether older adults are more likely to commit suicide than other age groups after adjusting for other possible causes. To explain the high suicide rate among older adults, we compared suicide rates between this section of the population and other age groups, rather than analyzing a specific age group. We used suicidal ideation as a risk factor for planning and attempting suicide [9], as well as for committing suicide.

Our purpose was to prospectively examine whether older adults are more likely to develop first-onset suicidal ideation (FoSI) than other age groups after adjusting for various factors, including those related to socioeconomic status (SES), health, the environment, and mental health. In addition, we suggest possible factors associated with higher suicide rates in the older adults than in young and middle-aged individuals.

## 2. Materials and Methods

### 2.1. Study Sample and Dataset

We obtained data from the Korean Welfare Panel Study (KoWePS) between 2011 and 2021. KoWePS, conducted by the Korean Institute of Health and Social Affairs (KIHASA) in conjunction with the Social Welfare Research Institute of Seoul National University, is an ongoing longitudinal study of a nationally representative sample of South Korean households for which data are collected annually [10]. Both the data and the study’s reference manual can be accessed through the KoWePS website (https://www.koweps.re.kr:442/main.do) Accessed on 23 May 2022. Trained interviewers conduct face-to-face interviews at participants’ residences using a structured survey questionnaire. The first KoWePS began in 2006, but information regarding suicidal ideation was not measured until the sixth wave in 2011. Thus, we used data from the sixth (2011) to sixteenth (2021) waves. In the sixth wave, 11,633 participants aged 19–89 years answered the survey questionnaire. Among them, we selected 9488 individuals who had never developed suicidal ideation. We excluded 360 individuals who could not complete the survey questionnaire because of absence, dementia, or mental retardation. Additionally, we excluded 440 individuals due to missing data, as well as 679 people who were either without follow-up after their baseline assessment or lost during follow-up and then returned. At baseline in 2011, 8061 individuals with no history of suicidal ideation remained in the study. The last follow-up wave was defined as the year of FoSI, loss to follow-up, or the eleventh wave, whichever occurred first.

### 2.2. Outcome Variable

The dependent variable was FoSI. At baseline (2011), we chose individuals who responded to the survey question “Until now, have you ever seriously thought about killing yourself?” and gave negative responses. During the follow-up waves (2012–2021), the development of FoSI was defined by a positive response to the survey question “Within the previous year, have you ever seriously thought about killing yourself?” among individuals with no history of suicidal ideation.

### 2.3. Explanatory Variable

To compare different ages, we categorized individuals into three age groups. We classified those aged 19–39 years as the “young” group, those aged 40–64 years as the “middle-aged” group, and those aged 65 or older as the “older adult” group. During the follow-up, we reclassified some individuals into an older age group when necessary.

### 2.4. Covariates

We selected diverse covariates related to suicidal ideation [11,12]. We measured covariates for each wave and grouped them under SES, health, environmental, health-related, and mental variables. To avoid reverse causation, we used covariates identified one year before each wave as covariates for each wave. 

Household-equalized disposable income, household net wealth, level of education, and type of labor force were classified as SES variables. Equalized disposable household income and net financial wealth were measured using the survey questionnaire. Household disposable income was defined as gross household income after deducting direct taxes and payment of social security contributions [1,10,13]. Gross income includes income from wages and salaries, self-employed income, realized property income, social benefits in cash, social insurance, or aid provided in the form of cash [1,10]. Household net wealth encompasses financial assets (savings, currency and deposits, and stocks), land and dwellings, agricultural machines, cars, and livestock. Household net financial assets were defined as the total value of a household’s financial worth or the sum of the overall financial and non-financial assets minus liabilities [1,10]. Household disposable income and household net wealth were equalized by dividing each item by the square root of the number of household members [14]; this figure was then divided into quartiles before the selection of participants each year. Education level was categorized as middle school, high school, and college or higher. The individual labor force was categorized as full-time permanent, precarious, self-employed, and not in the labor force.

The presence of disabilities, chronic ailments, hospital admissions in the past year, the score of the Alcohol Use Disorders Identification Test (AUDIT), and current smoking status were classified as health-related variables. The presence of chronic disease was defined as the administration of medication for at least 6 months. The AUDIT score was developed by the World Health Organization as a simple screening method for excessive drinking and to assist in a brief 10-question assessment of recent alcohol use, alcohol dependence symptoms, and alcohol-related problems [15]. In this study, an AUDIT score of eight or more was representative of moderate alcohol problems [16].

Marital status, the number of family members, and residential area were classified as environmental variables. Marital status was categorized as married, cohabiting, and other, including separation by death and unmarried individuals.

In this study, we assessed depressive symptoms using self-rated health status and assessment models such as the Rosenberg Self-Esteem Scale (RSES), which is based on an individual’s self-esteem that has a positive relationship with their psychological health, social adjustment, and quality of life. Self-esteem, in particular, plays a significant role in personal and social axes, especially when people have a sense of well-being [17]. In addition, the Rosenberg Self-Esteem Scale (RSES) and perceived health status were defined as the most proximal causes and intervening variables of suicidal ideation. The RSES establishes a positive or negative orientation toward oneself and is an overall evaluation of one’s worth or value [18]. The RSES is not only linked to suicidal ideation [19] but can also moderate the effects of life stressors on suicidal ideation [20]. In this study, we included the baseline and time-varying RSES as continuous variables, which ranged from 0 to 40.

### 2.5. Statistical Analysis

To identify statistically significant differences between groups, we performed a chi-square test to compare the categorical variables. We carried out analysis of variance to compare continuous variables. The dataset in this study was annually measured per person and observation for the longitudinal study. We detected first-onset events; we excluded records after the development of suicidal ideation from the observations of that person. Using this dataset of discrete-time survival analyses, we employed a generalized estimating equation (GEE) with exchangeable working correlations to estimate the odds ratios (ORs) and 95% confidence intervals (CIs) for the development of FoSI in young and middle-aged individuals compared with older adults. 

In this study, we set the reference model—in which only sex and follow-up year were considered—as the standard reference model for progressive adjustments. To identify key stressors capable of inducing suicidal ideation, we progressively adjusted selected groups of variables in the GEE analysis. We then compared changes in the statistical significance and strength of the association between the various models. A stressor model adjusted for SES-, health-, and environment-related stressors was the fully adjusted model. Lastly, we added mental health variables as moderators of stressors that are capable of inducing suicidal ideation to yield an overly adjusted model. The addition of both disposable household income level and net wealth level to the reference model, while stratifying by age group, revealed the relative importance of the economic stressors to be examined. All comparisons were two-tailed; we deemed comparisons with *p* values of less than 0.05 to be statistically significant. We performed all statistical analyses using SAS software (Version 9.4, SAS Institute Inc., Cary, NC, USA).

## 3. Results

Table 1 outlines the baseline characteristics of 2714 older adults, 3329 middle-aged, and 2018 young individuals with no history of suicidal ideation in 2011. The older adults exhibited the following characteristics compared to the other age groups: lower self-esteem, more depressive symptoms, a larger proportion of females, lower disposable household income, a lower level of education, less participation in the labor force, a higher prevalence of chronic disease and disability, more hospital admissions, a smaller number of family members, and a worse perception of their health status. During the ten-year follow-up period, the total person-years and follow-up periods were 60,104 and ten years, respectively. The total number of person-years and follow-up periods decreased annually from 8061 in 2012 to 4380 in 2021. The incidence of FoSI per 1000 person-year decreased steadily from 23.5 in 2012 to 7.3 in 2021. When categorized by age, the rate was higher in age groups ≥ 65 years. (Table 2). Among the 60,104 observations, 958 individuals developed FoSI, with an incidence rate (IR) of 15.9 per 1000 person-years. The IRs of the older adult, middle-aged, and young groups were 22.0, 13.0, and 11.3 per 1000 person-years, respectively.

Table 3 depicts the results of the discrete-time survival analyses in which we progressively adjusted the selected groups of variables. The reference model (Model 1), adjusted for sex and follow-up year, indicates that middle-aged (OR: 0.57, 95% CI: 0.50–0.66) and young (OR: 0.43, 95% CI: 0.35–0.53) individuals were less likely to develop FoSI than older adults. When we added the environmental, health-related, or socioeconomic variables to the reference model, the positive association between age group and the development of FoSI was attenuated but still statistically significant. In addition, when we added the socioeconomic and environmental, socioeconomic and health-related, or environmental and health-related variables to the reference model, the positive association between age group and the development of FoSI was attenuated but still statistically significant. However, in the stressor model, when we added socioeconomic, health-related, and environmental variables to the reference model, the statistically significant positive association between age group and the development of FoSI disappeared. Further, when we added mental health-related variables to the stressor model, the positive association in the reference model turned into a negative association in the full model.

Table 4 underscores the relative importance of the economic stressors examined by adding both disposable household income level and net wealth level to the reference model. Among the older adults, net household wealth was significantly associated with the incidence of FoSI, whereas disposable household income was an important predictor.

## 4. Discussion

We attempted to explain why older adults are more likely to develop FoSI than other age groups. A higher propensity to develop FoSI among older adults disappeared if their socioeconomic conditions became equal to those of the other age groups. Moreover, if their mental health status was equal to that of the other age groups, they were less likely to develop FoSI than the other age groups. Among socioeconomic factors, wealth among the older adults and income in the other age groups were key factors.

Traditionally, suicide rates rise with age and are the highest among older adults in many countries [21,22]. This trend is pronounced in Western nations with extended family structures and in Asian countries where older adults are culturally venerated [6,23]. During the Asian economic crisis of 1997–1998, the suicide rate in Asia rose substantially, especially among older adults [24]. Suicide rates of older adults in this study are similar to that of previous results. 

The disappearance of statistically significant differences between age groups in the model, where we adjusted selected variables progressively, suggests that some factors could explain the high suicide rates of older adults. A recent South Korean study reported that there were no statistically significant associations between income level and suicidal ideation in older adults; however, there was significantly higher suicidal ideation among young people with lower incomes [25]. Young and middle-aged adults are more likely to be directly influenced by changes in economic circumstances, such as income level, than older adults [24]. After adjusting for socioeconomic variables, the positive association between age and suicidal ideation was not observed. This finding implies that when SES is controlled for across groups, older adults are as likely to develop FoSI as those in other age groups. Therefore, this phenomenon indicates that SES might be an important predictor of a higher incidence of suicidal ideation in the older adult population. In 2008, the income poverty rate of South Koreans over 65 was 49.6% (the highest among OECD nations), which may be the result of the public pension scheme introduced in 1988 [2,26], as stated on the OECD website. Likewise, the older adults in this study had lower incomes than those in the other age groups. As we added the variables to the reference model, this relationship became negative. After we added mental health-related variables to the stressor model, this negative association was strengthened, signaling that if all adjusted values were equal, older adults would be more likely to develop FoSI than those in the other age groups. 

However, a few previous studies have also found results contrary to those of our study. For instance, a US study reported that among individuals aged 15 to 54 years, the highest risk of FoSI and suicide attempts occurs during the late teens and early 20s, and young individuals are more likely to develop FoSI after adjusting for SES [9,27]. A study of 17 countries revealed that, after adjusting for SES, other age groups were more likely to develop FoSI than those over 65 [6,7]. These studies demonstrated that after adjusting for SES and other covariates, older adults were less likely to develop suicidal ideation than those in other age groups. 

Our study has several limitations. First, the baseline participants with no history of suicidal ideation could have introduced bias due to the healthy survivor effect. As older adults have more opportunities to develop suicidal ideation during their longer lifetimes, they might have a stronger resistance to the development of suicidal ideation than other age groups. We observed this phenomenon in the strengthening of the negative association between age and FoSI after adjusting for mental health-related variables. Second, the follow-up period in this study was 10 years, which is not sufficient to detect FoSI. However, consistent with the fact that the suicide rate in South Korea is the highest in the world, a considerable number of suicide events occurred in the follow-up period. Third, we did not include risk factors for suicide and psychiatric disorders (except for depressive symptoms) in this study due to a lack of information in the KoWePS [28]. Fourth, we did not check the lack of statistical significance and loss of statistical power due to the multiple comparison problem while adding many variables in the equations. Particularly, we focused on suicide ideation in age groups. Accordingly, it is necessary to analyze an appropriate multiple testing correction to reduce false positives in future studies [29].

Despite these limitations, this study has several strengths. First, the design is longitudinal, with a 10-year follow-up period, and we excluded individuals with a history of suicidal ideation. Thus, the incident cases in this study were FoSI cases. Second, all variables in this study were time-dependent and measured annually. Hence, we considered changes in all variables in our analysis. Third, the KoWePS data that we used are representative of the South Korean population. Fourth, to the best of our knowledge, this study is one of the first to show that older adults are more likely to develop FoSI in a reference model, but less likely in adjusted models. This phenomenon is most likely due to the unique circumstances of a rapid rise in the suicide rate and the high prevalence of poverty among older adults in South Korea.

## 5. Conclusions

In summary, according to this prospective 10-year follow-up study, if stressors and mental health factors for suicidal ideation are similar, older adults will be less likely to develop FoSI than those in other age groups. Low stress is the primary factor that explains why older adults are more likely to develop FoSI than those in other age groups in a country where poverty among older adults is very high. Furthermore, we can consider combining this with possible reasons for the discrepancy in results, as well as suggestions for future research (that is, what subsequent research should focus on to address these discrepancies in the results). Future studies, in particular, should be prospective and not retrospective in design, so that all relevant information is available with enough follow-up periods.

## Figures and Tables

**Table 1:** Baseline characteristics of the participants at the beginning of follow-up in 2011.

Variables	Total		≥65 Years Old		40–64 Years Old		20–39 Years Old		*p* Value
	8061	(100.0)	2714	(33.7)	3329	(41.3)	2018	(25.0)	
Sex									<0.001
*Male*	3475	(43.1)	1065	(39.2)	1537	(46.2)	873	(43.3)	
*Female*	4586	(56.9)	1649	(60.8)	1792	(53.8)	1145	(56.7)	
Household income									0.98
*High*	2006	(24.9)	666	(24.5)	836	(25.1)	504	(25.0)	
*Mid-high*	2039	(25.3)	685	(25.2)	836	(25.1)	518	(25.7)	
*Mid-low*	2013	(25.0)	695	(25.6)	824	(24.8)	494	(24.5)	
*Low*	2003	(24.9)	668	(24.6)	833	(25.0)	502	(24.9)	
Household wealth									0.67
*High*	1978	(24.5)	672	(24.8)	809	(24.3)	497	(24.6)	
*Mid-high*	2049	(25.4)	681	(25.1)	833	(25.0)	535	(26.5)	
*Mid-low*	2027	(25.2)	674	(24.8)	839	(25.2)	514	(25.5)	
*Low*	2007	(24.9)	687	(25.3)	848	(25.5)	472	(23.4)	
Education level									<0.001
*College or higher*	2281	(28.3)	161	(5.9)	754	(22.7)	1366	(67.7)	
*High school*	2307	(28.6)	306	(11.3)	1374	(41.3)	627	(31.1)	
*Middle school*	3473	(43.1)	2247	(82.8)	1201	(36.1)	25	(1.2)	
Labor force									<0.001
*Full-time permanent*	1797	(22.3)	37	(1.4)	886	(26.6)	874	(43.3)	
*Precarious*	1310	(16.3)	212	(7.8)	735	(22.1)	363	(18.0)	
*Self-employed*	1905	(23.6)	820	(30.2)	929	(27.9)	156	(7.7)	
*Not in the labor force*	3049	(37.8)	1645	(60.6)	779	(23.4)	625	(31.0)	
Disability									<0.001
*No*	7354	(91.2)	2337	(86.1)	3040	(91.3)	1977	(98.0)	
*Yes*	707	(8.8)	337	(13.9)	289	(8.7)	41	(2.0)	
Admitted to the hospital									<0.001
*No*	7148	(88.7)	2248	(82.8)	3063	(92.0)	1837	(91.0)	
*Yes*	913	(11.3)	466	(17.2)	266	(8.0)	181	(9.0)	
Chronic disease									<0.001
*No*	4440	(55.1)	455	(16.8)	2125	(63.8)	1860	(92.2)	
*Yes*	3621	(44.9)	2259	(83.2)	1204	(36.2)	158	(7.8)	
AUDIT score									<0.001
*Non-drinker*	3677	(45.6)	1742	(64.2)	1319	(39.6)	616	(30.5)	
*0–7*	2844	(35.3)	709	(26.1)	1200	(36.1)	935	(46.3)	
*8 or more*	1540	(19.1)	263	(9.7)	810	(24.3)	467	(23.1)	
Current smoker									<0.001
*No*	5244	(65.1)	1750	(64.5)	2084	(62.6)	1410	(69.9)	
*Yes*	2817	(35.0)	964	(35.5)	1245	(37.4)	608	(30.1)	
Marital status									<0.001
*Married and cohabiting*	5560	(69.0)	1669	(61.5)	2772	(83.3)	1119	(55.5)	
*Other*	2501	(31.0)	1045	(38.5)	557	(16.7)	899	(44.6)	
Number of family members									<0.001
*Three or more*	4641	(57.6)	541	(19.9)	2466	(74.1)	1634	(81.0)	
*Two*	2374	(29.5)	1455	(53.6)	671	(20.2)	248	(12.3)	
*One*	1046	(13.0)	718	(26.5)	192	(5.8)	136	(6.7)	
Residential area									<0.001
*Rural*	2020	(25.1)	1062	(39.1)	687	(20.6)	271	(13.4)	
*Urban*	6041	(74.9)	1652	(60.9)	2642	(79.4)	1747	(86.6)	
Perceived health status									<0.001
*Bad*	4182	(51.9)	1256	(46.3)	566	(17.0)	105	(5.2)	
*Moderate*	1952	(24.2)	866	(31.9)	798	(24.0)	288	(14.2)	
*Good*	1927	(23.9)	592	(21.8)	1965	(59.0)	1625	(80.5)	
Rosenberg Self-Esteem Scale [*M, (SD)*]	30.7	(4.0)	28.9	(4.1)	31.1	(3.7)	32.4	(3.3)	<0.001
CES-D-11 [M, (SD)]	3.8	(4.6)	5.7	(5.4)	2.9	(3.7)	2.4	(3.5)	<0.001

Unit: number of participants (percentage). AUDIT, Alcohol Use Disorders Identification Test; *M*, mean; *SD*, standard deviation.

**Table 2:** Crude incidence rate of first-onset suicidal ideation according to age group.

	Total			≥65 Years Old			40–64 Years Old			20–39 Years Old		
Calendar	Events	Person-	Incidence	Events	Person-	Incidence	Events	Person-	Incidence	Events	Person-	Incidence
Year		Years	Rate		Years	Rate		Years	Rate		Years	Rate
2012	189	8061	23.5	93	2857	32.6	71	3354	21.2	25	1850	13.5
2013	185	7503	24.7	90	2732	32.9	68	3214	21.1	27	1557	17.3
2014	205	6955	29.5	116	2639	44.0	72	3033	23.7	17	1283	13.3
2015	88	6462	13.6	51	2500	20.4	29	2895	10.0	8	1067	7.5
2016	77	6016	12.8	49	2386	20.5	21	2742	7.7	7	888	7.9
2017	52	5663	9.2	35	2315	15.2	14	2610	5.4	3	738	4.1
2018	51	5380	9.5	25	2267	11.0	19	2494	7.6	7	619	11.3
2019	44	5053	8.7	27	2189	12.3	13	2382	5.5	4	482	8.2
2020	35	4631	7.6	16	2099	7.6	16	2169	7.4	3	363	8.3
2021	32	4380	7.3	16	2075	7.7	14	2032	6.9	2	273	7.3
Total	958	60,104	15.9	518	24,059	22.0	337	26,925	13.0	103	9120	11.3

Incidence rate: events per 1000 person-years.

**Table 3:** The association between age and first-onset suicidal ideation by progressive adjustment: Results of the generalizing estimating equation analysis.

Models	≥65 Years Old	40–64 Years Old			20–39 Years Old		
	Reference	OR	95% CI		OR	95% CI	
Crude model	1.00	0.58	0.50	0.66	0.52	0.42	0.64
Reference model (Model 1) = crude model + sex + year	1.00	0.57	0.50	0.66	0.43	0.35	0.53
Model 2 = reference model + environmental variables	1.00	0.77	0.65	0.92	0.54	0.43	0.60
Model 3 = reference model + health-related variables	1.00	0.83	0.71	0.97	0.79	0.61	1.03
Model 5 = reference model + socioeconomic variables	1.00	0.82	0.69	0.98	0.73	0.55	0.97
Model 6 = reference model + socioeconomic + environmental variables	1.00	0.93	0.77	1.14	0.82	0.60	1.12
Model 7 = reference model + socioeconomic + health-related variables	1.00	0.94	0.79	1.13	0.95	0.70	1.28
Model 4 = reference model + environmental + health-related variables	1.00	1.00	0.84	1.20	0.89	0.68	1.18
Stressor model (Model 8) = reference model + socioeconomic + health-related + environmental variables	1.00	1.01	0.83	1.24	1.01	0.73	1.39
Full model (Model 9) = stressor model + mental health variables	1.00	1.68	1.35	2.11	1.94	1.38	2.73

OR: odds ratio; CI: confidence interval.

**Table 4:** The importance of economic stressors, stratified by age group.

Variables	≥65 Years Old				40–64 Years Old				20–39 Years Old			*P* Trend
	OR	95% CI		*P* Trend	OR	95% CI		*P* Trend	OR	95% CI		
Household income												
*High*	1.00			<0.001	1.00			<0.001	1.00			<0.001
*Mid-high*	1.15	0.85			1.98	1.27			1.64	0.82		
*Mid-low*	1.48	1.10			2.69	1.72			1.15	0.53		
*Low*	1.71	1.27			3.79	2.47			3.31	1.64		
Household wealth												
*High*	1.00			<0.001	1.00			<0.001	1.00			0.570
*Mid-high*	1.28	0.95			0.71	0.48			0.69	0.37		
*Mid-low*	1.54	1.14			0.74	0.51			0.57	0.28		
*Low*	2.50	1.87			1.53	1.09			1.19	0.65		

Sex, calendar year, household income, and household wealth are included in this model.

## Data Availability

Publicly available datasets were analyzed in this study. This data can be found here: https://www.koweps.re.kr:442/main.do/ Accessed on 23 May 2022.

## References

[1] Raschke N., Mohsenpour A., Aschentrup L., Fischer F., Wrona K.J. (2022). Socioeconomic factors associated with suicidal behaviors in South Korea: Systematic review on the current state of evidence. BMC Public Health.

[2] OECD (2021). Health at a Glance 2021: OECD Indicators.

[3] Korean National Statistical Office (2020). Death and Cause of Death in Korea.

[4] Andrés A.R. (2005). Income inequality, unemployment, and suicide: A panel data analysis of 15 European countries. Appl. Econ..

[5] Chiu H.F., Dai J., Xiang Y.T., Chan S.S., Leung T., Yu X., Hou Z.J., Ungvari G.S., Caine E.D. (2012). Suicidal thoughts and behaviors in older adults in rural China: A preliminary study. Int. J. Geriatr. Psychiatry.

[6] Nock M.K., Borges G., Bromet E.J., Alonso J., Angermeyer M., Beautrais A., Bruffaerts R., Chiu W.T., de Girolamo G., Gluzman S. (2008). Cross-national prevalence and risk factors for suicidal ideation, plans and attempts. Br. J. Psychiatry.

[7] Pritchard C., Hansen L. (2005). Comparison of suicide in people aged 65–74 and 75+ by gender in England and Wales and the major Western countries 1979–1999. Int. J. Geriatr. Psychiatry.

[8] Scocco P., de Girolamo G., Vilagut G., Alonso J. (2008). Prevalence of suicide ideation, plans, and attempts and related risk factors in Italy: Results from the European Study on the Epidemiology of Mental Disorders—World Mental Health Study. Compr. Psychiatry.

[9] Kessler R.C., Borges G., Walters E.E. (1999). Prevalence of and risk factors for lifetime suicide attempts in the National comorbidity Survey. Arch. Gen. Psychiatry.

[10] The Korean Institute of Health and Social Affairs (2021). User’s Guide the 11th Wave of the Korean Welfare Panel Study.

[11] Conwell Y., Duberstein P.R., Hirsch J.K., Conner K.R., Eberly S., Caine E.D. (2010). Health status and suicide in the second half of life. Int. J. Geriatr. Psychiatry.

[12] Manthorpe J., Iliffe S. (2010). Suicide in later life: Public health and practitioner perspectives. Int. J. Geriatr. Psychiatry.

[13] OECD (2011). Society at a Glance 2011: OECD Social Indicators.

[14] Förster M.F., D’Ercole M.M. (2012). The OECD Approach to Measuring Income Distribution and Poverty: Counting the Poor—New Thinking About European Poverty Measures and Lessons for the United States.

[15] Babor T.F., Higgins-Biddle J., Saunders J., Monteiro M. (2001). The Alcohol Use Disorders Identification Test—Guidelines for Use in Primary Care.

[16] Miller W.R. (1995). Motivational Enhancement Therapy Manual: A Clinical Research Guide for Therapists Treating Individuals with Alcohol Abuse and Dependence.

[17] Park J.-Y., Park E.-Y. (2019). The Rasch Analysis of Rosenberg Self-Esteem Scale in Individuals with Intellectual Disabilities. Front. Psychol..

[18] Rosenberg M. (1989). Society and the Adolescent Self-Image.

[19] De Man A.F., Gutiérrez B.I.B. (2002). The relationship between level of self-esteem and suicidal ideation with stability of self-esteem as moderator. Can. J. Behav. Sci. Rev. Can. Sci. Comport..

[20] Wilburn V.R., Smith D.E. (2005). Stress, self-esteem, and suicidal ideation in late adolescents. Adolescence.

[21] Hawton K., van Heeringen K. (2009). Suicide. Lancet.

[22] Shah A., De T. (1998). Suicide and the elderly. Int. J. Psychiatry Clin. Pract..

[23] Lau B.W.K., Pritchard C. (2001). Suicide of older people in Asian societies: An international comparison. Australas. J. Ageing.

[24] Chang S.S., Gunnell D., Sterne J.A., Lu T.H., Cheng A.T. (2009). Was the economic crisis 1997–1998 responsible for rising suicide rates in East/Southeast Asia? A time-trend analysis for Japan, Hong Kong, South Korea, Taiwan, Singapore and Thailand. Soc. Sci. Med..

[25] Lee H.Y., Hahm M.I., Park E.C. (2013). Differential association of socio-economic status with gender- and age-defined suicidal ideation among adult and elderly individuals in South Korea. Psychiatry Res..

[26] OECD (2013). Pensions at a Glance 2013: OECD and G20 Indicators.

[27] Kessler R.C., Berglund P., Borges G., Nock M., Wang P.S. (2005). Trends in suicide ideation, plans, gestures, and attempts in the United States, 1990–1992 to 2001–2003. JAMA.

[28] Cavanagh J.T., Carson A.J., Sharpe M., Lawrie S.M. (2003). Psychological autopsy studies of suicide: A systematic review. Psychol. Med..

[29] Noble W.S. (2009). How does multiple testing correction work?. Nat. Biotechnol..

